# ^68^Ga-labeled fibroblast activation protein inhibitor (FAPI) PET/CT for locally advanced or recurrent pancreatic cancer staging and restaging after chemoradiotherapy

**DOI:** 10.7150/thno.95329

**Published:** 2024-07-08

**Authors:** Giulia Metzger, Christian Bayerl, Julian MM Rogasch, Christian Furth, Christoph Wetz, Marcus Beck, Felix Mehrhof, Holger Amthauer, Pirus Ghadjar, Christopher Neumann, Uwe Pelzer, Daniel Zips, Frank Hofheinz, Jane Grabowski, Imke Schatka, Sebastian Zschaeck

**Affiliations:** 1 Charité - Universitätsmedizin Berlin, corporate member of Freie Universität Berlin and Humboldt-Universität zu Berlin, Department of Nuclear Medicine, Berlin, Germany.; 2 Charité - Universitätsmedizin Berlin, corporate member of Freie Universität Berlin and Humboldt-Universität zu Berlin, Department of Radiology, Berlin, Germany.; 3 Charité - Universitätsmedizin Berlin, corporate member of Freie Universität Berlin and Humboldt-Universität zu Berlin, Department of Radiation Oncology, Berlin, Germany.; 4 Charité - Universitätsmedizin Berlin, corporate member of Freie Universität Berlin and Humboldt-Universität zu Berlin, Department of Hematology, Oncology and Tumor Immunology, Berlin, Germany.; 5 Charité - Universitätsmedizin Berlin, German Cancer Consortium (DKTK), partner site Berlin, and German Cancer Research Center (DKFZ), 69120, Berlin, Heidelberg, Germany.; 6 Institute for Radiopharmaceutical Cancer Research, Helmholtz-Zentrum Dresden-Rossendorf, Dresden, Germany.; 7 Charité - Universitätsmedizin Berlin, corporate member of Freie Universität Berlin and Humboldt-Universität zu Berlin, Berlin Institute of Health (BiH), 10117 Berlin, Germany.

**Keywords:** locally recurrent pancreatic adenocarcinoma, locally advanced pancreatic adenocarcinoma, fibroblast activation protein inhibitor, positron emission tomography, radiochemotherapy

## Abstract

**Purpose:** ^68^Ga-labeled fibroblast activation protein inhibitor (FAPI) is a novel PET tracer with great potential for staging pancreatic cancer. Data on locally advanced or recurrent disease is sparse, especially on tracer uptake before and after high dose chemoradiotherapy (CRT). The aim of this study was to evaluate [^68^Ga]Ga-FAPI-46 PET/CT staging in this setting.

**Methods:** Twenty-seven patients with locally recurrent or locally advanced pancreatic adenocarcinoma (LRPAC n = 15, LAPAC n = 12) in stable disease or partial remission after chemotherapy underwent FAPI PET/CT and received consolidation CRT in stage M0 with follow-up FAPI PET/CT every three months until systemic progression. Quantitative PET parameters SUV_max_, SUV_mean_, FAPI-derived tumor volume and total lesion FAPI-uptake were measured in baseline and follow-up PET/CT scans. Contrast-enhanced CT (ceCT) and PET/CT data were evaluated blinded and staged according to TNM classification.

**Results:** FAPI PET/CT modified staging compared to ceCT alone in 23 of 27 patients in baseline, resulting in major treatment alterations in 52% of all patients (30%: target volume adjustment due to N downstaging, 15%: switch to palliative systemic chemotherapy only due to diffuse metastases, 7%: abortion of radiotherapy due to other reasons). Regarding follow-up scans, major treatment alterations after performing FAPI PET/CT were noted in eleven of 24 follow-up scans (46%) with switch to systemic chemotherapy or best supportive care due to M upstaging and ablative radiotherapy of distant lymph node and oligometastasis. Unexpectedly, in more than 90 % of the follow-up scans, radiotherapy did not induce local fibrosis related FAPI uptake.

During the first follow-up, all quantitative PET metrics decreased, and irradiated lesions showed significantly lower FAPI uptake in locally controlled disease (SUV_max_ p = 0.047, SUV_mean_ p = 0.0092) compared to local failure.

**Conclusion:** Compared to ceCT, FAPI PET/CT led to major therapeutic alterations in patients with LRPAC and LAPAC prior to and after radiotherapy, which might help identify patients benefiting from adjustments in every treatment stage. FAPI PET/CT should be considered a useful diagnostic tool in LRPAC or LAPAC before and after CRT.

## Background

Ductal adenocarcinoma of the pancreas (PDAC) is the most common cancer of the pancreas. With a 5-year survival rate below 10%, PDAC is also one of the most aggressive types of cancer 1. Due to late onset of symptoms that occur primarily in advanced stages, only 10-20% of patients qualify for curatively intended surgical resection 2. Both locally advanced pancreatic adenocarcinoma (LAPAC) and locally recurrent pancreatic adenocarcinoma (LRPAC) are usually unresectable. The outcome for these patients is dismal with median overall survival ranging between five and eleven months 3. Best treatment approaches for these patients remain controversial; recommendations include chemotherapy, fractionated image-guided radiotherapy (IGRT) or stereotactic radiotherapy. For tumors with unfavorable biology, especially those with development of distant metastases, patients often receive initial chemotherapy and radiotherapy only in the case of tumor control (i.e. partial remission or stable disease). The approach of performing IGRT only in the clinical setting of tumor control has shown some promising results in retrospective analyses 4, 5. However, optimal utilization of IGRT in LRPAC und LAPAC is controversial due to the high prevalence of distant metastases and the risk associated with radiosensitivity of surrounding organs 4, 5.

In radiotherapy treatment planning, the administration of highly conformal treatment doses to the tumor while minimizing radiation doses to surrounding healthy tissue is crucial and could be ensured by multimodal imaging. However, the use of [^18^F]FDG PET/CT in PDAC is not routinely recommended by clinical guidelines 6. High physiological FDG uptake in some organs (e.g., the liver and spleen) and accompanying inflammation might contribute to low diagnostic specificity and affect diagnosis or tumor delineation of PDAC in case of concomitant pancreatitis or tumor-related congestion 7. An alternative, more specific radiopharmaceutical with low uptake in adjacent organs in the upper abdomen and reliably high tumor uptake by PDAC is therefore required to improve loco-regional and whole-body staging. The typical abundance of desmoplastic stroma in PDAC makes it an ideal candidate for PET imaging with ligands that target the fibroblast activation protein (FAP) 8. In contrast to normal fibroblasts, cancer-associated fibroblasts (CAFs) contribute to tumor growth, invasion and metastasis via crosstalk with neoplastic cells 9.

^68^Ga-labeled fibroblast activation protein inhibitor (FAPI) is a promising new molecular ligand in PET imaging that targets FAP, which is overexpressed in CAFs. Several studies suggest that PET/CT and PET/MRI with ^68^Ga-labeled 8, 10-14 and ^18^F-labeled 15-17 FAPI could be superior to [^18^F]F-FDG PET/CT in the detection of PDAC, in particular regarding the higher sensitivity and accuracy 14, 15 as well as an excellent TBR (tumor-to-background-ratio) and the detection of lymph node and distant metastases 16, 17. Still, most published data are on primary diagnosed PDAC prior to any surgical intervention. Data on recurrent or locally advanced pancreatic cancer in the setting of radiation oncology treatment is limited. To the best of our knowledge to date only one retrospective study published data on the use of [^68^Ga]Ga-FAPI PET/CT in a sample of 19 patients 18. The same research group published data demonstrating that [^68^Ga]Ga-FAPI PET/CT could facilitate target definition and increase consistency in radiation oncology in pancreatic cancer 19. Another retrospective analysis evaluated the impact of [^68^Ga]Ga-FAPI PET/CT on radiotherapy planning in 14 patients with PDAC 20. No data is available on follow-up FAPI PET/CT imaging after radiation in locally advanced or locally recurrent pancreatic cancer.

The purpose of this study was therefore to investigate the impact of [^68^Ga]Ga-FAPI-46 PET/CT on diagnostic performance in staging and re-staging patients with LAPAC or LRPAC prior to and after radiotherapy. Furthermore, this study aims to evaluate a potential benefit of quantitative PET metrics predicting early treatment response.

## Materials and Methods

### Patient cohort

The patient cohort consisted of 27 patients (12 male, 15 female) between 42 and 87 years of age with histologically confirmed PDAC. Inclusion criteria were adequate data of previous radiological imaging (ceCT and/or abdominal MRI), showing stable disease or partial remission and cM0-situation after receiving capecitabine-based chemotherapy, not more than three months between radiological staging and FAPI PET/CT, an Eastern Cooperative Oncology Group (ECOG) status 21 of less than three, and consent to radiation therapy. Exclusion criteria were previous abdominal radiation therapy or evidence of progression between radiological imaging and FAPI PET/CT. All patients with LRPAC had undergone previous surgery (Whipple surgery n = 11, pancreatectomy n = 2, distal pancreatectomy n = 1, duodenopancreatectomy n = 1). All patients with LRPAC underwent chemotherapy in an adjuvant setting after surgery. Patients with LAPAC underwent chemotherapy as initial therapy with FOLFIRINOX (folinic acid, fluorouracil, irinotecan, and oxaliplatin). Patients were given a range of 3-13 therapeutic cycles. Patients underwent restaging imaging (abdominal MRI in combination with ceCT of the chest [n = 4] or low dose CT of the chest [n = 3], or ceCT of the chest and the abdomen [n = 20] alone) and, in case of stable disease or remission, were reevaluated by the interdisciplinary tumor board of upper intestinal malignancies of the Charité university hospital to determine candidacy for consolidating chemoradiotherapy (CRT). All patients scheduled for CRT between March 2022 and August 2023 received pretreatment imaging with [^68^Ga]Ga-FAPI-46 PET/CT and combined irradiation planning CT. After completion of CRT, patients were scheduled for follow-up clinical visits every three months, including an assessment of B-symptoms, general symptoms, physical examination and dynamic of tumor markers, followed by [^68^Ga]Ga-FAPI-46 PET/CT imaging. Follow-up FAPI PET/CT imaging was discontinued in case of disseminated metastases. Data collection was carried out in a retrospective study design. Restaging after adjuvant chemotherapy for evaluation of CRT was completed a median of 36 days before the FAPI PET/CT scans.

Patient characteristics are summarized in **Table 1**. Individual patient data is shown in **Supplementary Table S1**.

### [^68^Ga]Ga-FAPI-46 PET/CT imaging

Synthesis and labeling of [^68^Ga]Ga-FAPI-46 was prepared following an established protocol published by Spreckelmeyer *et al.* 22. The precursor was obtained from SOFIE (Dulles, VA, USA). The injected activity ranged from 155 to 200 MBq [median 181 MBq, 4.8 mCi]. A Philips Gemini TF 16 scanner with time of flight (TOF) capability (Philips Astonish TF technology) was used for PET/CT imaging. PET scans were obtained after a median of 74 minutes [range 58-132 min] post-injection of the radiopharmaceutical. A time point of 60 minutes post-injection has been proven to be suitable for a high tumor-to-background ratio (TBR) 23-26. For irradiation treatment planning and target delineation, baseline [^68^Ga]Ga-FAPI-46 PET/CT was performed with an additional 4D planning CT and vacuum mattress, and PET/CT imaging was performed with the patient in supine radiotherapy treatment position. In total, 51 [^68^Ga]Ga-FAPI-46 PET/CTs (27 baseline and 24 follow-up scans) were obtained according to previously published studies 18. The [^68^Ga]Ga-FAPI PET/CT always included a non-enhanced low-dose CT for attenuation correction. Due to previously performed ceCT scan within 4 weeks or contraindications for contrast agent (renal failure or previous allergic reactions), no contrast agent was administered in six scans of baseline imaging, affecting six patients. In all other cases, a diagnostic ceCT scan on arterial and venous phase imaging was acquired as well (automated tube current modulation; maximum tube current-time product, 200 mAs; tube voltage, 120 kV; delay after contrast agent injection, 80 seconds; bolus rate, 3 mL/s). PET raw data were reconstructed using iterative reconstruction (ordered subset expectation maximization; OSEM) with time of flight (TOF) analysis (BLOB-OS-TF; iterations, 3; subsets, 33; filter, 'smooth' [kernel width, 14.1 cm; relaxation parameter, 0.7]; matrix, 144 × 144; voxel size, 4.0 × 4.0 × 4.0 mm^3^). PET data were corrected for randoms, scatter, attenuation and dead time.

### Image evaluation

The ceCT of the PET/CT was reviewed retrospectively without the PET data by two experienced, board-certified radiologists (>six years and >15 years of experience). In six cases of baseline imaging, a contrast agent was not administered due to previously performed ceCT scan within four weeks or contraindications for contrast agent (renal failure or allergic reactions). For cases where a contrast agent could not be administered, low dose CT data in combination with previously performed ceCT or MRI scans were used for evaluation. Radiological imaging evaluation was performed blinded to the previously collected clinical data, such as CT, MRI, EUS or histopathological results. The complete dataset of the [^68^Ga]Ga-FAPI-46 PET/CT scans was evaluated by one board-certified nuclear physician and one resident nuclear physician (>15 years and >two years of experience). The ceCT data and the full PET/CT dataset were both evaluated at a dedicated workstation (Visage Client Software version 7.1.18.6265, San Diego, CA, USA) in a standard clinical setting of the department of nuclear medicine in Charité university hospital. Staging was performed according to the 8^th^ edition of the UICC classification 27. Changes in the TNM classification and alterations in therapeutic management were recorded and discussed with an experienced, board-certified radiation oncologist (>15 years of experience).

In case of inconsistent findings in [^68^Ga]Ga-FAPI-46 PET/CT scans, the case was reevaluated by the interdisciplinary tumor board and biopsy or further imaging (e.g., MRI) was performed. Abortion of radiotherapy (i.e., switch to palliative intended systemic chemotherapy alone, watch and wait, best supportive care) or change of the target volume were defined as major therapy alterations. Modulations in the intensity of radiation were defined as minor changes. If available, histopathology or radiographic follow-up were used as references for the diagnostic performance of [^68^Ga]Ga-FAPI PET/CT.

FAPI-derived tumor volume (FDTV), total lesion FAPI-uptake (TLF), SUV_max_ and SUV_mean_ of primary tumors or recurrent tissue were obtained using dedicated software (ROVER version 2.1.20, ABX, Radeberg, Germany) as previously described 28. FDTV was used in terms of MTV (metabolic tumor volume). TLF was calculated by multiplying SUV_mean_ by FDTV. Volumes of interest (VOI) of the locally recurrent tissue or the primary pancreatic tumor were delineated using a background-adapted threshold-based algorithm relative to the maximum activity in the lesion and calculated automatically if SUV_max_ surpassed a standardized threshold value within the tumor region of 41%. The definition of VOIs and calculation of the diameter of the locally recurrent tissue or the primary tumor were performed in consensus by two nuclear medicine physicians.

### Radiation treatment

Radiation was planned as follows: the primary target volume consisted of the tumor, the corresponding peripancreatic lymph nodes and suspicious or initially affected abdominal lymph nodes with a clinical target volume and PTV margins with a single dose of 1.7 Gray (Gy) in 30 fractions. Macroscopic tumor volumes as defined by standard imaging, PET information and 4D-CT were boosted by a simultaneous integrated boost (SIB) with 2 Gy single dose with reduced PTV margins of 5 millimeters (mm) and subtraction of critical organs at risk (OARs) (i.e., duodenum/ stomach and small bowel). A further dose escalation of the boost volume was performed using a simultaneous integrated protection approach. Additionally, a further safety margin of 4 mm was calculated around the critical OARs. This volume was subtracted from the boost volume and further boosted with a single dose SIB of 2.2 Gy. The total radiation doses to the target volumes were therefore 51, 60 and 66 Gy. Alterations of target volume delineation by PET were documented for evaluation. Concomitant to radiotherapy, patients received capecitabine 830 mg per m² in the morning and evening of each radiotherapy treatment, according to Mukherjee *et al.* 29.

### Statistical analysis

The PET parameters in paired patient groups (i.e., baseline PET vs. follow-up PET or progressive disease yes vs. no) were compared using a two-sided Mann-Whitney U test. Statistical significance was assumed at a p-value of less than 0.05. Delta values were defined as following: fractional differences of the parameters in follow-up and baseline data were computed as difference of follow-up value and baseline value divided by baseline value and are expressed in percent of the baseline value. This reads for e.g. ∆ FDTH



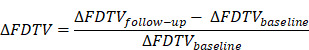



Delta values for the other parameters were computed accordingly and are expressed in percent. Statistical analysis was performed with the R language and environment for statistical computing Version 4.3.1. (R Core Team. R: A Language and Environment for Statistical Computing. Vienna, Austria: R Foundation for Statistical Computing; 2023).

## Results

### Diagnostic performance

#### Baseline [^68^Ga]Ga-FAPI-46 PET/CT

**Figure 1:**
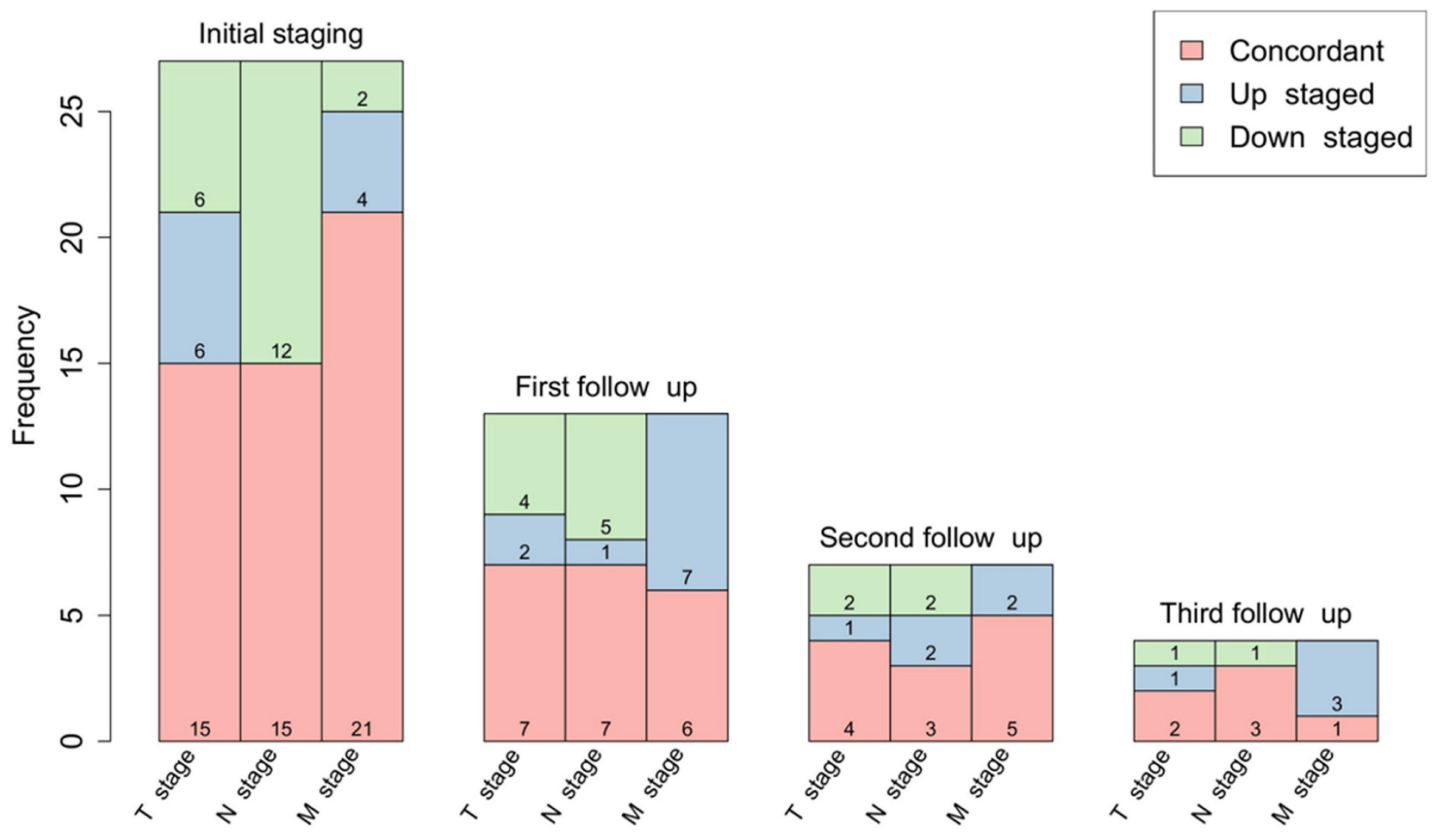
Changes in TNM staging based on differences between blinded reading of FAPI-PET/CT and CT scans before treatment and during first, second and third follow-up.

The baseline [^68^Ga]Ga-FAPI-46 PET/CT staging before planned CRT resulted in changes of TNM in 23 of 27 patients (85%). **Figure 1** shows differences between restaging imaging and FAPI PET/CT at each point in time. **Supplementary Table S2** shows detailed and individual changes in TNM staging.

##### T staging at baseline

In 12 of 27 (44%) cases, an up- or downstaging of the local tumor extension (T stage) was noted, which led to major treatment modifications for four patients, which are described as follows. In one patient with LAPAC of the pancreatic head, extensive cross-organ FAPI-tracer distribution was detected, suggestive of duodenal infiltration, which was corroborated by an additional MRI. Radiotherapy was omitted in this patient to avoid radiogenic intestinal damage.

In one patient suffering from LRPAC, locally recurrent tumor tissue close to the hepatic portal was suspected based on the detection of an unclear lesion in the restaging CT scan after primary chemotherapy. As no FAPI signal of the tissue could be detected in the subsequently performed [^68^Ga]Ga-FAPI PET/CT, the lesion was interpreted as avital scar tissue following previous chemotherapy. This assumption was further supported by the absence of significant size changes in follow-up CT scans. Instead of the initially considered local radiotherapy, further close follow-up and watchful waiting was recommended. No disease relapse was detected up to one year later.

Based on the CT scans at baseline [^68^Ga]Ga-FAPI-46 PET/CT, two patients with suspected LRPAC were staged T0 N0 M0. FAPI signaling allowed the detection of discrete suspicious tissue infiltrating the mesenteric root, which was considered to be an inconclusive finding based on radiological imaging alone. As vital recurrent tumor tissue could not be ruled out, each patient qualified for local ablative radiotherapy.

In general, extensive tracer distribution of the whole pancreas in patients (especially those with LAPAC) suffering from chronic obstruction of the pancreatic duct challenged the correct delineation of local tumor extension.

##### N staging at baseline

**Figure 2:**
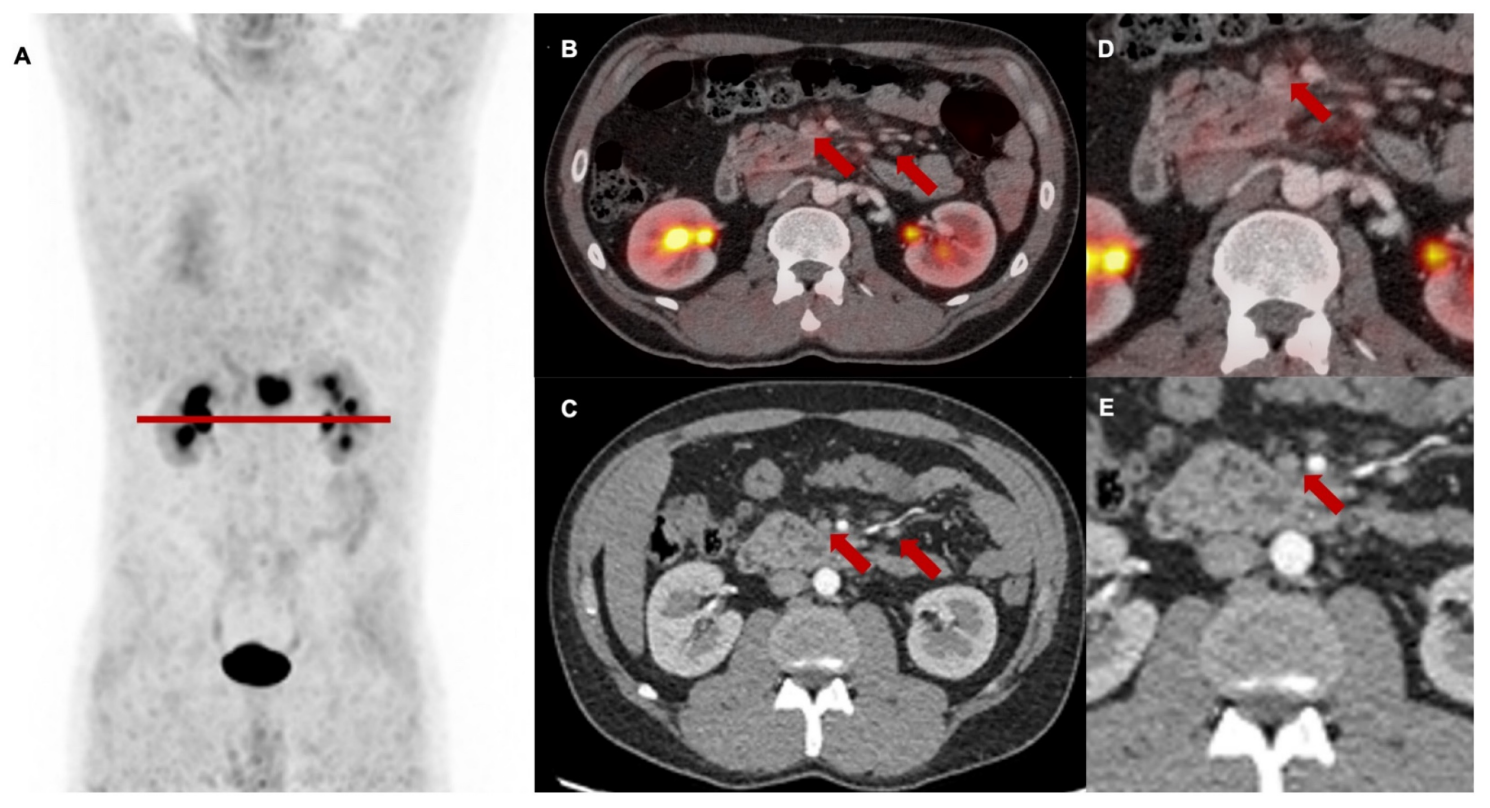
Baseline [^68^Ga]Ga-FAPI-46 PET/CT of a patient with locally recurrent pancreatic adenocarcinoma at the mesentery root. **A** maximum intensity projection with a red line representing axial slices B-E, **B and D** transaxial fusion of PET/CT data of the upper abdomen, **C and E** transaxial*,* corresponding contrast-enhanced CT scan in arterial phase. Suspicious peripancreatic lymph nodes with a short axis diameter over 10 mm (red arrows) without FAPI signal were rated benign and consequently downstaged.

Regarding N stage, FAPI-PET led to a downstaging of morphologically suspicious peripancreatic and mesenteric lymph nodes from N1 or N2 to N0 in 12 of 27 cases (44%). In eight cases (30%), these findings resulted in a change of the target volume as these lymph nodes were not included in the irradiation field. This N downstaging by [^68^Ga]Ga-FAPI-46 PET/CT was confirmed by stable absence of increasing lymph node diameters during follow-up and by the lacking evidence for tumor progression within the clinical assessment of patients after radiotherapy every three months (example given in **Figure 2**).

##### M staging at baseline

Alteration of the M stage was noted in six of 27 patients (22%). Upstaging to cM1 influenced the therapeutic approach in four of 27 patients (15%). In these cases, FAPI PET/CT imaging revealed distant metastases, which were not detected by morphological imaging alone. Two patients were upstaged from M0 to M1 due to intensive FAPI expression of a discretely thickened peritoneal sheet (suspected peritoneal carcinomatosis). The other two patients were classified M1 based on evaluation of CT data alone, but intense FAPI uptake helped identify additional bone and liver metastases in the absence of morphologic CT findings. Consequently, these patients were excluded from radiotherapy and received palliative intended systemic chemotherapy instead. M downstaging occurred in two patients. In one case, atypically located pancreatic recurrent tissue was misinterpreted as an adrenal metastasis. In another patient with suspected liver metastases on ceCT, FAPI signaling of multiple liver segments was attributed to repeated draining of congestion-related liver abscesses. This suspected diagnosis was confirmed by the medical history of the patient and the concomitant evidence of increased inflammatory markers.

**Figure 3:**
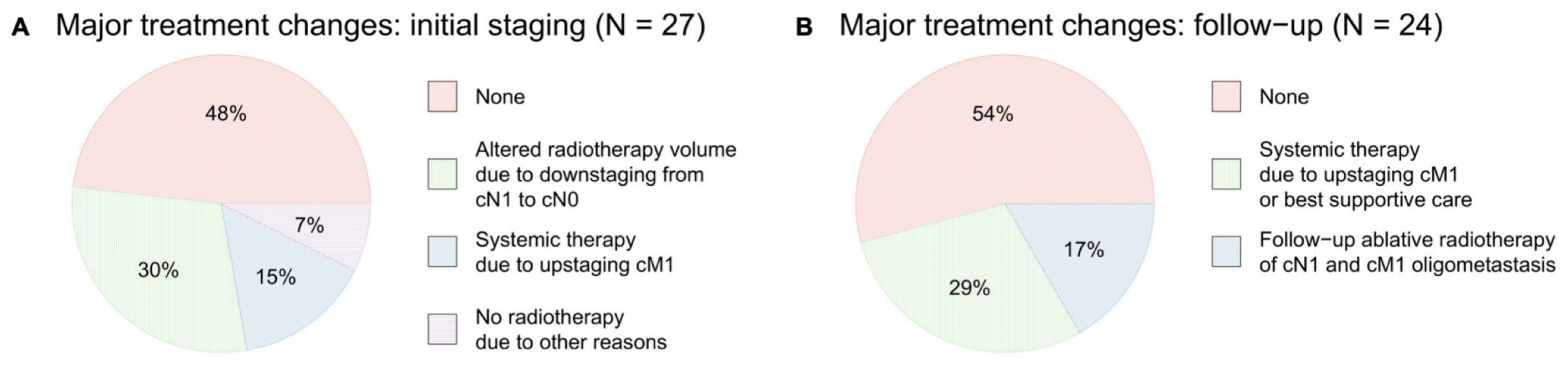
** A** major treatment changes after baseline [^68^Ga]Ga-FAPI-46 PET/CT, **B** major treatment changes after follow-up [^68^Ga]Ga-FAPI-46 PET/CT (1^st^, 2^nd^ and 3^rd^ follow-up combined).

In conclusion, baseline [^68^Ga]Ga-FAPI-46 PET/CT led to major changes in the therapeutic approach in 14 of 27 patients (52%), including alterations of the target volume in case of lymph node downstaging in eight of 27 patients (30%) omission of local radiotherapy due to diffuse metastases in four of 27 patients (15%) and the abortion of radiotherapy due to other reasons (e.g., infiltration of surrounding organs or no measurable vital tumor tissue) in two of 27 patients (7%) Additional diagnostic measures (abdominal MRI) were taken in one patient (4%) to verify duodenal infiltration. Additional details are provided in **Table 2 and in Figure 3A**.

#### Follow-up [^68^Ga]Ga-FAPI-46 PET/CT

The first post-treatment follow-up scans after three months were performed in 13 patients, second follow-up scans in seven patients and a third follow-up scan in four patients. Details regarding the differences in staging between CT and PET/CT reading are provided in **Figure 1. Supplementary Table S2** shows detailed and individual changes in the TNM classification.

##### T staging at follow-up

**Figure 4:**
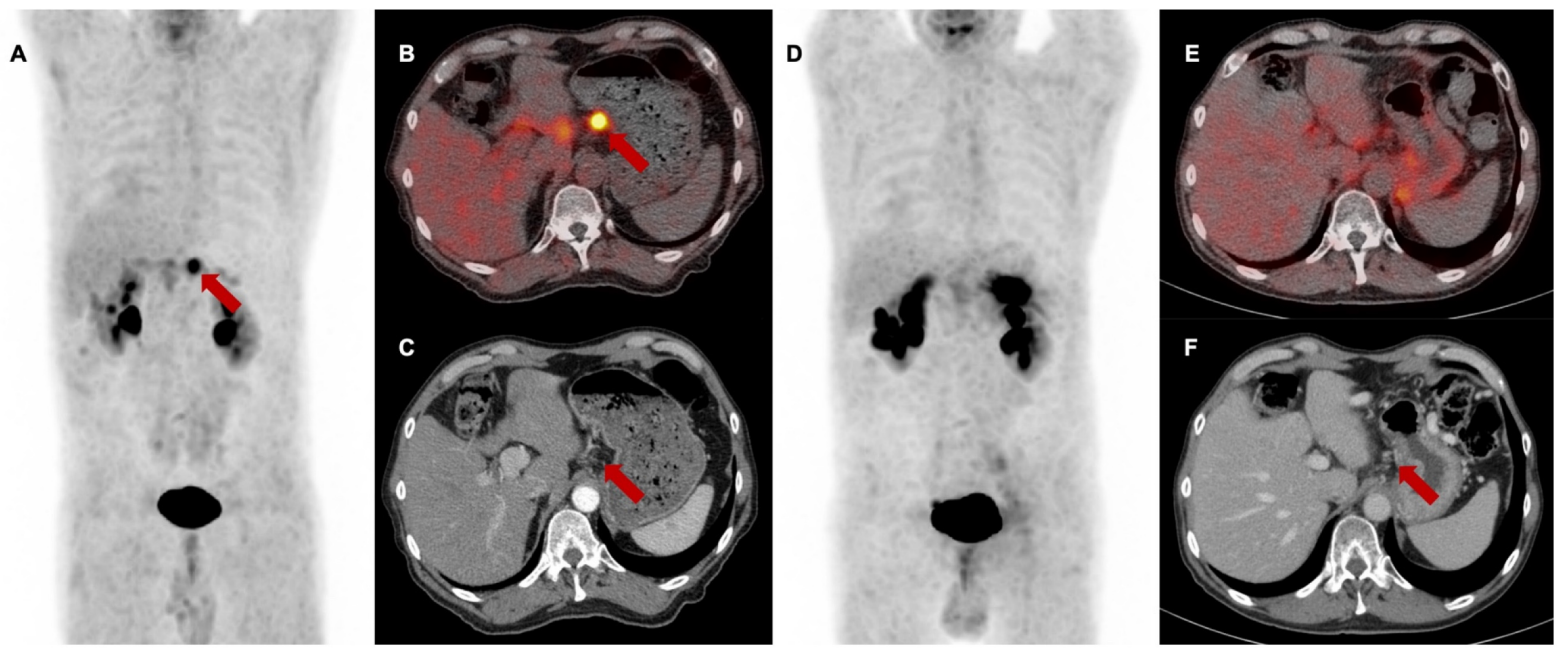
[^68^Ga]Ga-FAPI-46 PET/CT of a patient with locally recurrent pancreatic adenocarcinoma before (Baseline, **A-C**) and 3 months after (first follow-up **D-F**) treatment with chemoradiotherapy (Patient #7). **A and D** maximum intensity projections, **B and E** transaxial fusion of PET/CT of the upper abdomen, **C and F** transaxial contrast-enhanced CT scan of the upper abdomen. Red arrows (**A-C**) mark intense FAPI uptake of small-volume tumor tissue at the minor gastric margin. The red arrow in **F** marks stable tumor tissue without measurable FAPI signaling post-treatment. SUV_max_ at 1^st^ follow-up was 8,2 / SUV_mean_ was 5,0 and at 2^nd^ follow-up 1,7 / 1,4, respectively. In the absence of the detection of vital tumor remnants in the [^68^Ga]Ga-FAPI-46 PET/CT, complete remission of the disease was assumed. In this case, the significant decrease of the FAPI uptake in the 1^st^ follow-up scan was associated with local tumor control within the next 6 months after therapy. Patient #7 showed local progression in the 3^rd^ follow-up scan 9 months post CRT.

Local tumor control (i.e., stable disease or remission) after high dose chemoradiotherapy was achieved in 5 of 13 patients (38%) with mostly concordant T classification or minor variations. Here, reduced FAPI expression correlated with either reduced tumor volume or no measurable lesion (example shown in **Figure 4**).

One female patient with LAPAC of the pancreatic head was staged T4 both through analysis of post-treatment CT-data and combined PET/CT-data. Increasing and a rather diffuse FAPI tracer uptake and distribution of the irradiated tumor and the previously inserted duodenal stent challenged the differentiation between postradiogenic fibrosis and tumor proliferation. Endoscopic biopsy excluded a malignant origin and showed granulocytic inflammatory infiltrate consistent with postradiogenic duodenitis. Notably, inconclusive findings concerning T staging due to post-treatment fibrosis induction did not occur in any other case of follow-up scans.

##### N staging at follow-up

Similar to the baseline PET/CT scans, many cases (53% (7/13) at 1^st^ follow-up, 29% (2/7) at 2^nd^ follow-up and 25% (2/4) at 3^rd^ follow-up) of suspicious lymph nodes in the CT did not present a relevant FAPI expression, resulting in a high rate of N downstaging in 46% (11/24). However, in one patient with LRPAC, intense FAPI signaling enabled the identification of an occult cervical distant lymph node metastasis, which was confirmed by biopsy, resulting in a major treatment alteration via local radiotherapy of this distant metastasis.

##### M staging at follow-up

**Figure 5:**
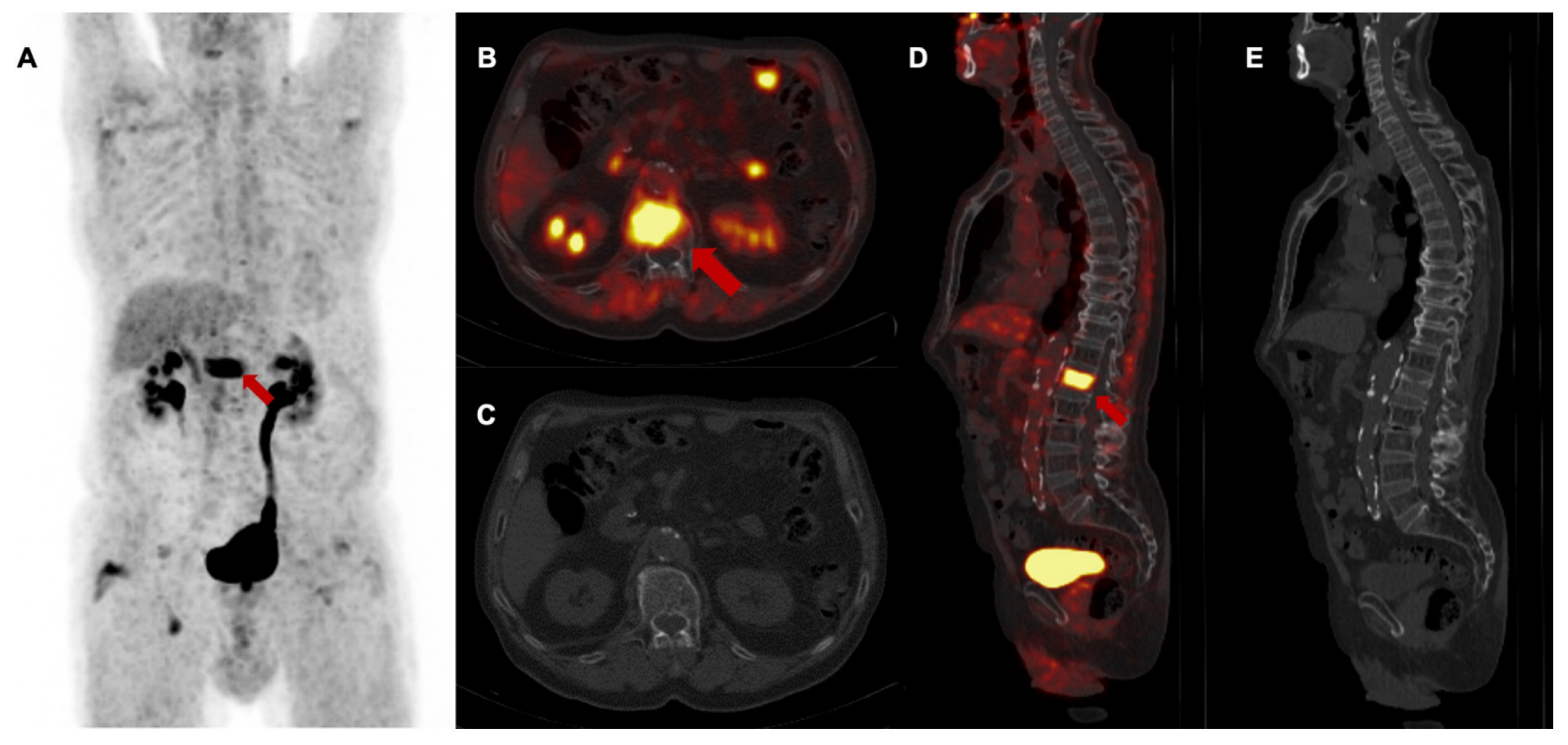
3^rd^ follow-up [^68^Ga]Ga-FAPI-46 PET/CT of a patient with locally recurrent pancreatic adenocarcinoma 9 months after chemoradiotherapy (Patient #2) **A** maximum intensity projection, **B and D** transaxial and sagittal PET/CT fusion, **C and E** contrast-enhanced CT scan (bone window). Red arrows (**A, B, D**) mark intense FAPI signal of first lumbar vertebra without a detectable lesion in the bone. In the absence of any morphological finding in the CT scan, this scan was classified as M upstaging and, in the consensus of an interdisciplinary tumor board, considered as singular metastatic spread to the bone. Biopsy and histopathological confirmation were not performed. The patient received curative intended local radiotherapy of the first lumbar vertebra.

Follow-up [^68^Ga]Ga-FAPI-46 PET/CT showed a high rate of M upstaging, including a change of classification from M0 to M1 or the detection of different metastatic lesions compared to CT alone in seven of 13 patients (53%) at first follow-up, in two of seven patients (29%) at second follow-up and in three of four patients (75%) at third follow-up. Intense FAPI uptake without a measurable lesion in the CT scan was suspicious for metastatic spread at the following sites: 44% peritoneum, 25% liver, 13% soft tissue, 8% bone, brain and lung. In total, additional diagnostic measures were taken in five of 24 scans (21%) (5/24), including mainly liver and brain MRIs. In one patient with LRPAC, who maintained local tumor control up to the third follow-up scan, diffuse FAPI signaling of a lumbar vertebra with unremarkable bone structure occurred and was staged as oligometastatic spread to the bone, which was followed by ablative radiotherapy (**Figure 5**). After radioablation, the follow-up scans showed a significant decrease in FAPI uptake, indicating treatment response of the initial osseus tumor burden.

In total, major treatment changes in consideration of the PET/CT data at follow-up imaging occurred in eleven of 24 scans (46%) including a switch to systemic therapeutic approach or best supportive care in three of 13 patients (23%) at first follow-up, in two of seven patients (29%) at second follow-up and in two of four patients (50%) at third follow-up, and additional ablative radiotherapy of cN1 or cM1 oligometastases in two of 13 patients (15%) at first follow-up, 14% at second follow-up and in one of four patients (25%) at third follow-up. Additional diagnostic measures (e.g., brain or liver MRI, lymph node biopsy) were taken in five of 24 (21%) follow-up scans. Additional details are provided in **Table 2 and Figure 3B.**

### Pre- and posttreatment evaluation of quantitative PET parameters

The biodistribution of [^68^Ga]Ga-FAPI-46 was determined in terms of SUV_max_, SUV_mean_, TLF and FDTV.

**Figure 6:**
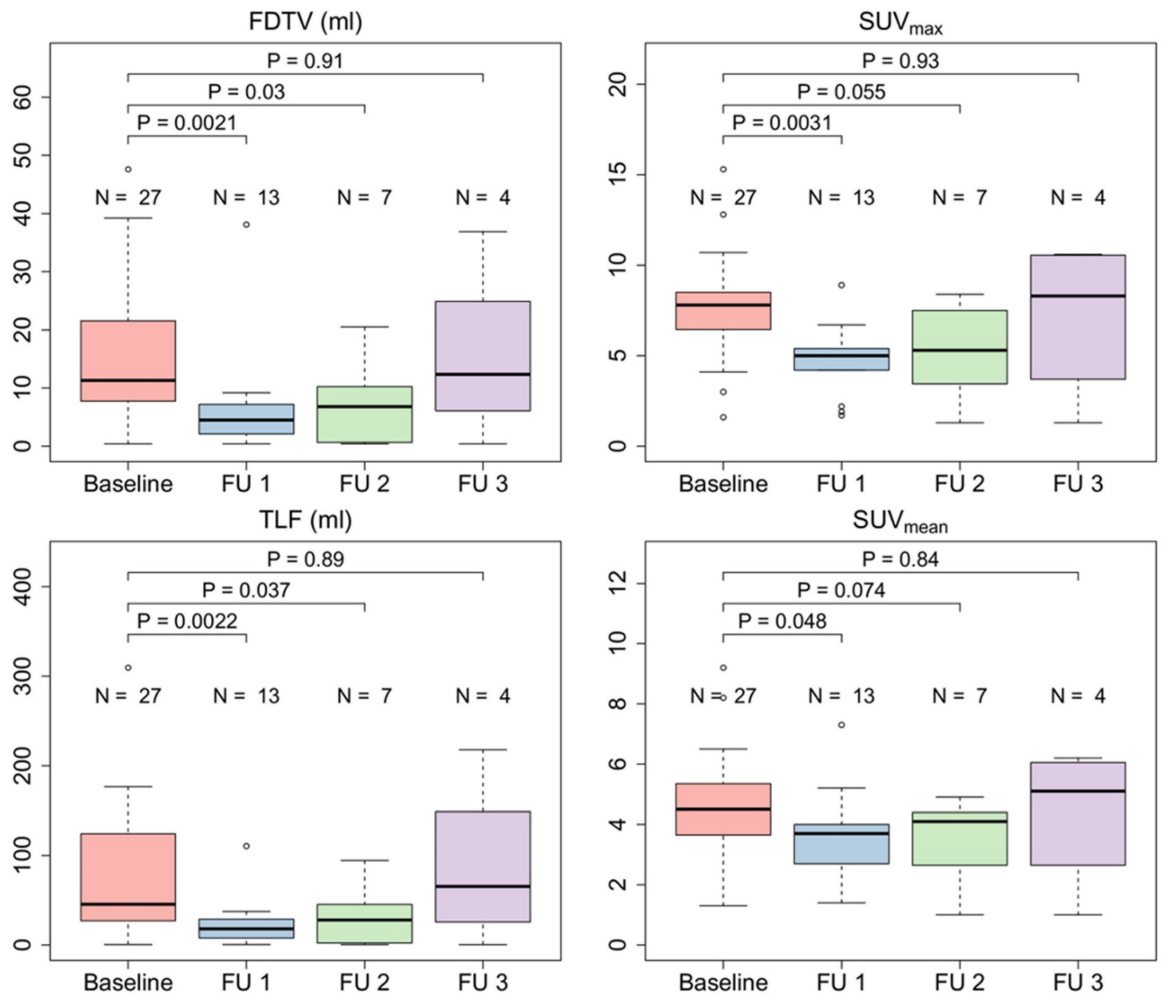
Quantitative FAPI-PET parameters FAPI-derived tumor volume (FDTV), SUV_max_, SUV_mean_, and total lesion FAPI-uptake (TLF) of all irradiated patients (n = 27) before treatment and during 1^st^, 2^nd^ and 3^rd^ follow-up.

Radiation led to a significant reduction of the SUV_max_ (median 7.7 vs. 5.5, p = 0.0012), FDTV (median 11.3 vs. 4.5, p = 0.027) and TLF (median 45.4 vs. 18.2, p = 0.0018) in patients three months after CRT treatment. In patients with additional follow-up scans, quantitative parameters seemed to increase thereafter and reached similar average values as at the baseline scan in the sample of patients with a third follow-up scan. **Figure 6** shows the course of quantitative PET parameters in all patients.

As LAPAC is highly radioresistant and PET might provide information about local progression, quantitative FAPI PET/CT parameters before treatment and at first follow-up examination after three months were compared between patients without local progression at six months post-treatment (four of seven patients) versus patients with early recurrence within six months (three of seven patients). In the first follow-up scan three months post-treatment, only one patient showed local progression, whereas 12 patients presented either stable disease, partial or complete remission.

**Figure 7:**
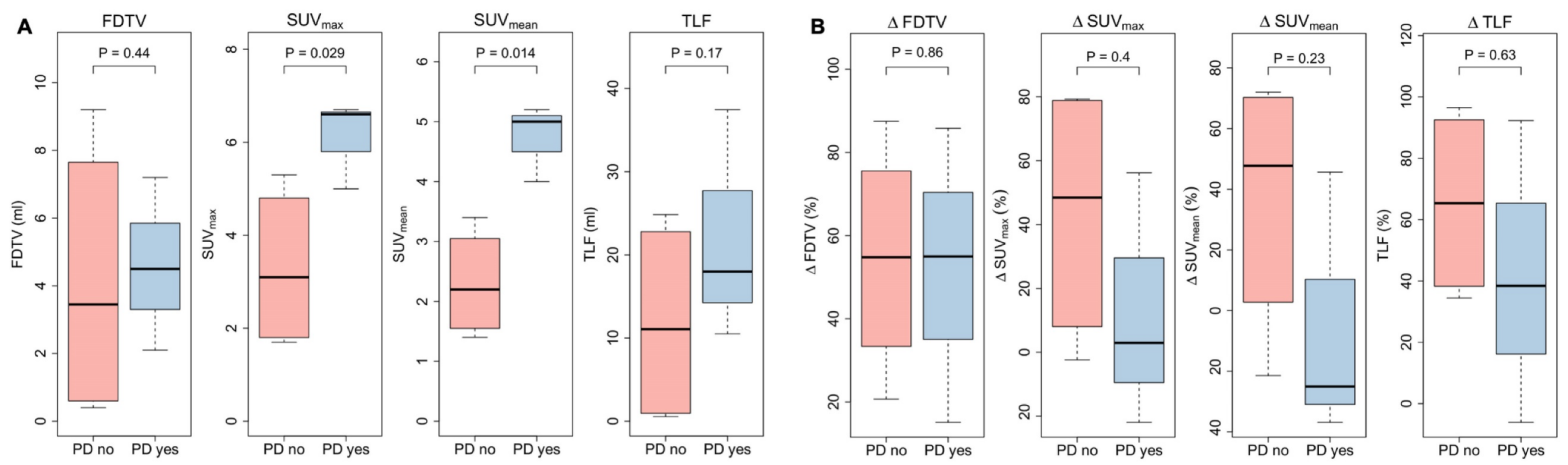
**A** quantitative FAPI-PET parameters FAPI-derived tumor volume (FDTV), SUV_max_, SUV_mean_, and total lesion FAPI-uptake (TLF) three months after chemoradiation**. B** delta values before treatment and three months after treatment of quantitative PET parameters**.** Patients without local progression up to six months after treatment (PD no, 4/7) were compared with patients who experienced early local progression (PD yes, 3/7).

Patients without local progression within the irradiated volume showed significantly lower SUV_max_ and SUV_mean_ three months after CRT (SUV_max_ 3.1 vs. 6.6, p = 0.047, and SUV_mean_ 2.2 vs. 5.5, p = 0.0092), while FDTV and TLF did not show significant differences (FDTV p = 0.86; TLV p = 0.38). Delta values between baseline and first follow-up scan of SUV_max_, SUV_mean_, FDTV and TLF did not seem to be associated with local control. Corresponding box plots are shown in **Figure 7**.

## Discussion

Accurate diagnostic procedures are needed to ensure the successful treatment of highly aggressive PDAC. We investigated the potential benefit of [^68^Ga]Ga-FAPI-46 PET/CT compared to ceCT alone in patients suffering from LRPAC or LAPAC before and after undergoing CRT. As FDG PET/CT has several limitations in staging PDAC, and is not part of the guidelines in pancreatic cancer staging 6, evaluation of the benefits of FAPI PET/CT towards ceCT imaging seemed reasonable. For example, in 64 patients with primarily diagnosed PDAC a head-to-head comparison of ^68^Ga-FAPI versus ^18^F-FDG or contrast-enhanced CT showed a higher detection rate of tumors with ^68^Ga-FAPI on a per-lesion (84.7% vs. 46.5% vs. 52.9%), per-patient (97.4% vs. 73.7% vs. 92.1%), or per-region (32.6% vs. 18.8% vs. 23.7%) basis, resulting in minor and major treatment challenges in 8% of the patients 14. Another monocentric, prospective study including seven patients with PDAC undergoing primary staging or restaging stated, that [^18^F]FAPI-74 detected 22% more lesions compared with [^18^F]FDG, especially with a better TBR (tumor-to-background-ratio) and visual lesion delineation 15. Furthermore, [^68^Ga]Ga-FAPI-04 outdated [^18^F]F-FDG in the preoperative prognostic performance in 32 patients with histologically confirmed PDAC. Here, pathologically enlarged tumor size, poor differentiation, and perineural invasion were associated with increased [^68^Ga]Ga-FAPI-04 uptake, but not with [^18^F]F-FDG uptake 30. Thus, to the best of our knowledge, the current study presents by far the largest dataset of patients with LRPAC or LAPAC that underwent [^68^Ga]Ga-FAPI-46 PET/CT imaging in this specific setting. This analysis suggests an additional value of [^68^Ga]Ga-FAPI-46 PET/CT in LRPAC and LAPAC patients undergoing CRT compared to standard-of-care ceCT imaging only.

As reported in a previous study including 51 patients with various tumor entities, [^68^Ga]Ga-FAPI presented a rather high and selective tumor uptake in PDAC and its metastases, resulting in an excellent TBR (tumor-to-background ratio) 24. Our findings are in line with another recently published retrospective analysis of the clinical impact of FAPI PET/CT on various tumor entities for radiotherapy planning or for addressing inconclusive findings in other imaging modalities. The study involved a large cohort of 226 patients in different clinical settings and included initial staging, follow-up imaging and assessment of metastatic spread. Here, major alterations in oncologic management were demonstrated due to changes in TNM stage, particularly for PDAC, lung, and head and neck cancers 31.

In the present study, FAPI-imaging influenced TNM staging and resulted in major treatment changes, especially for T staging in LRAPC. In two of 15 patients with LRPAC (13%), intense FAPI signaling of suspicious locally recurrent tissue close to the mesenteric root allowed clarification of inconclusive CT scan results and suggested vital tumor remnants rather than scar tissue, supporting the decision for performing adjuvant radiotherapy. Given the high sensitivity of [^68^Ga]Ga-FAPI imaging 14, FAPI PET/CT could be a potential method for detecting early recurrent tissue in post-treatment follow-up scans outside of the realm of radiotherapy planning. Additionally, scar tissue could be identified by the absence of intense FAPI uptake and verified by follow-up imaging where no size progression was detected within one year. Similar findings were previously reported in one patient with suspected local recurrence after undergoing Whipple surgery followed by FOLFIRINOX and a CT-based stage T2. The patient was downstaged after FAPI PET/CT as no vital recurrent tissue in the absence of FAPI signaling could be defined 18. Similar to [^18^F]F-FDG, FAPI could be regarded as a viability marker for tumor tissue in case of inconclusive radiological findings. Implementation of FAPI PET/CT in further clinical studies is recommended to evaluate the potential benefit, especially for patients with LRPAC.

Despite this, determining the T stage and local tumor extension for radiotherapy planning remains challenging using pre- and post-treatment imaging alone, especially for LAPAC. Previous studies on PDAC describe the occurrence of tumor-related congestion and build-up of exocrine secretion, resulting in pancreatitis, which complicates the differentiation of chronic inflammation and tumor mass in FAPI PET-scans. Even though a higher SUV_max_ and SUV_mean_ was observed in PDAC compared to pancreatitis, a certain overlap of uptake intensities was described after one hour of static imaging. Repeated imaging 10 minutes, 60 minutes and 180 minutes post injection showed a slight increase in FAPI-uptake of PDAC, whereas intensity of FAPI decreased in inflammatory tissue, indicating differential uptake kinetics 18. Another study suggested the additional value of multiple, early time point FAPI PET-scans ranging from 10 to 58 minutes, facilitating the differentiation between PDAC and pancreatitis via slightly increasing FAPI uptake of tumor lesions and decreasing tracer intensity of benign tissue 32. These results are in line with a previously published study by Pang *et al.* 12, where delayed imaging after three hours helped to distinguish between pancreatic cancer and pancreatitis in six of 12 patients with PDAC, in which intense FAPI uptake was observed throughout the whole pancreas. In contrast to patients with malignant tissue, FAPI signaling decreased significantly in individuals with pancreatitis 12. In our study, correct tumor delineation due to FAPI uptake throughout the whole pancreas in baseline and follow-up imaging was complicated in one of 12 patients (8%) with LAPAC. In this patient, two additional biopsies of the irradiated PDAC and the margins close to a duodenal stent, suspicious of local recurrence, could rule out a mass of malignant origin. Given the non-invasiveness, delayed imaging for determining T stage should be the favored procedure in LAPAC and concomitant pancreatitis. Furthermore diffusion-weighted MRI could facilitate tumor delineation and distinction between malignant and inflammatory tissue 33.

Interestingly, a very high rate of N downstaging was observed in this cohort. An improvement in the detection of lymph node metastases in pancreatic adenocarcinoma by [^68^Ga]Ga-FAPI-PET/MRI was reported by Zhang *et al.* 11, where 33% more lymph nodes with intense FAPI uptake were staged as metastatic compared to [^18^F]FDG-PET/CT, resulting in upstaging with [^68^Ga]Ga-FAPI-PET/MRI in four of 15 cases (27%). Therefore, this investigation may be the first to highlight the value of pretreatment baseline [^68^Ga]Ga-FAPI-46 PET/CT in downstaging suspicious regional lymph nodes and the clinical implications of altered target volume in subsequent radiotherapy.

With respect to distant metastases, we observed changes in up- and downstaging. In 15% of baseline [^68^Ga]Ga-FAPI-46 PET/CT and up to 50% of follow-up scans, distant metastases were not identified by CT data reading alone, with most cases of M upstaging in the follow-up scans attributed to peritoneal carcinomatosis (44%) and liver metastases (25%), respectively. This discrepancy between baseline and follow-up scans may be related to the fact that most of the patients were already staged cM0 by standard-of-care radiological imaging within a median of 36 days before undergoing [^68^Ga]Ga-FAPI-PET/CT. These significant alterations in M staging, when compared to CT scans alone, are in line with previously published analyses, where FAPI-PET-imaging led to upstaging in the majority of cases (nine of 12, 75%) in patients with recurrent or progressive PDAC 18. When compared to [^18^F]F-FDG PET/CT, earlier studies largely confirm the superiority of [68Ga]Ga-FAPI PET/CT 12-14, which is in line with studies utilizing 18F-labeled FAPI 15, 16. Zhang *et al.* 11 reported challenges in the detection of liver metastasis by [^68^Ga]Ga-FAPI-04 compared to [^18^F]F-FDG PET while this was mitigated by combined MRI. Still, other studies showed high detection rates of small liver metastasis 24, 34 or peritoneal carcinomatosis 35 by FAPI PET/CT in patients with various types of cancer. In this regard, further studies with focus on the detection of liver metastasis and distinction to fibrotic findings in PDAC patients, especially considering false positive findings due to chronic inflammation caused by congestion-related cholangitis or liver abscesses, are needed. Additionally, delayed imaging could be a helpful tool, although the decrease of FAPI signaling in hepatic bile duct was not statistically significant 12.

In our study, we confirmed the superiority of FAPI PET/CT when compared to CT alone. This may be relevant for the detection of lesions in the early stage of the metastatic process and potentially prevent rapid disease progression by allowing for the administration of early systemic therapy. Apart from changes of the therapeutic approach to systemic chemotherapy, [^68^Ga]Ga-FAPI PET/CT might help identify patients suitable for local ablative radiotherapy in case of oligometastases. In our study, the detection of soft tissue, bone and distant lymph node metastases enabled local radiotherapy in three of 27 patients (11%) and contributed to pain relief and overall tumor control.

Additionally, we present the first report on changes of quantitative FAPI PET metrics after high dose radiotherapy. Interestingly, we see a decrease of FAPI uptake three months after treatment in the whole cohort and a slight increase thereafter. More importantly, a first exploratory analysis revealed that marked decrease of SUV_max_ or SUV_mean_ might be associated with favorable local tumor control. This finding is of great interest, since it might identify patients that might benefit from early continuation of chemotherapy or other measures.

Furthermore, it is intriguing that FAPI uptake decreases after radiotherapy, since radiation is known to be a strong inducer of fibrosis. In that regard, preclinical experiments showed that FAP is upregulated after irradiation of cancer-associated fibroblasts 36. Given these observations, the decrease of FAPI uptake observed in our cohort of patients is especially remarkable because a pronounced decrease seemed to be associated with favorable local response to radiotherapy.

In general, in patients with significant volume reduction of tumor tissue, the partial volume effect needs to be taken into account, as it might result in an underestimation of tumor tracer uptake and lower values for SUV_max_ and SUV_mean_. Still, in this study, measurement of quantitative PET metrics might not have been influenced by the partial volume effect. In all patients with LAPAC, irradiated tumors did not show a size reduction above 50%, whereas irradiated locally recurrent tissue either presented a stable expansion in most of cases or showed morphologically complete remission.

The association of quantitative follow-up FAPI PET metrics with outcome is in line with published data of patients who received chemotherapy 37. In 47 patients treated with chemotherapy, Zhu *et al.* 30 reported initially defined tumor volume (termed MTV here within) and ΔMTV to be associated with survival and response to treatment. Another study on 37 surgically resected patients reported initial SUV_max_ to be associated with recurrence-free survival. Contrary to these findings, we did not observe an association of any initial quantitative PET metric with treatment response six months after CRT. This might be due to the small sample size or differing patient characteristics of our cohort, but could also indicate different response in irradiated patients. Furthermore, contrary to patients treated with chemotherapy only, we did not see a significant association of ΔFDTV in patients with response compared to non-responders. This might be, at least partially, explained by the problems of correct FDTV/MTV delineation. FDTV/MTV delineation can be challenging before treatment, especially in patients with additional fibrosis of the pancreas. This might further contribute to difficulties in diagnostic performance, as mentioned above. Even more difficult is the delineation of remnant tumor tissue after radiotherapy, especially as uptake of FAPI was significantly reduced and lesion-to-background-ratio is often relatively poor. Our data seem to indicate that SUV_max_ und SUV_mean_ might be more reliable and potentially promising parameters than volumetric values in this setting.

### Limitations

Study limitations included sample size, follow-up time/study length, and lack of histopathological validation.

While the existing study cohort was rather small, it still presents the highest number of subjects in a clinical setting of this nature. The rather short follow-up period of one year after radiotherapy might contribute to limited conclusions in terms of partly insignificant results in patient outcome. Therefore, prospective longitudinal studies with larger patient cohorts should be considered. To further assess the patients benefits and improvement of life quality after treatment alterations based on FAPI PET/CT imaging, multicentric study design seems to be crucial.

Lack of histopathological validation in cases of inconsistent findings between FAPI PET/CT and CT is a major shortcoming of the current study. This limitation particularly applies to suspiciously configured abdominal lymph nodes being downstaged by [^68^Ga]Ga-FAPI-46 PET/CT and cM1-upstaging of occult or unsuspicious lesions in the CT-data. However, performing invasive biopsy is not a clinical standard in advanced-stage pancreatic cancer, and some abdominal lesions, like lymph nodes in the retroperitoneal space, might not be amenable to biopsy. In addition, given a poor general condition due to therapy related side effects, the majority of the patients were not suitable for such an intervention. This limitation necessitated the use of alternative diagnostic measures to evaluate tumor progression. Consequently, patients were clinically evaluated every three months, providing a subject assessment of disease progression. Additionally, the dynamics of tumor markers, specifically carcinoembryonic antigen (CEA) and carbohydrate antigen 19-9 (CA 19-9) were monitored as surrogate parameters. These markers can reflect tumor burden and activity, offering a non-invasive method to track disease status. Symptom assessment, although valuable, is inherently subjective and may be influenced by various patient-specific factors, including individual pain threshold and psychological state. Nevertheless, combining these methods provided a comprehensive approach to monitor tumor progression in cases where histopathological confirmation was unattainable.

Still, given a good general status and sufficient compliance, histopathological examination was performed in a subset of lesions. A biopsy was sought particularly at unusual sites for metastasis. In one patient biopsy of a suspicious distant lymph node metastasis in the cervical region was, confirmed PDAC. However, biopsy of inconclusive lesions remains gold-standard and serves as the most suitable tool to verify metastatic spread in case of intensive FAPI signaling.

As discussed above, repeated and delayed imaging should be integrated into the FAPI PET/CT examination protocol to facilitate interpretation of FAPI-positive lesions, especially in radiation-naïve LAPAC. Furthermore, diffusion-weighted MRI should be considered a valuable diagnostic method to differentiate between pancreatic malignancy and inflammation.

## Conclusion

In patients suffering from LRPAC and LAPAC, pretreatment and follow-up [^68^Ga]Ga-FAPI PET/CT demonstrated benefits in the assessment of disease extent and diagnostic performance compared to radiological imaging alone. Alterations in TNM staging, especially N downstaging and M upstaging, led to major changes in the therapeutic approach in 46% of the cases, resulting in systemic therapy, local ablative radiotherapy in case of oligometastasis or watchful waiting. Given the superiority of FAPI PET/CT especially in PDAC, implementing [^68^Ga]Ga-FAPI PET/CT in the diagnostic algorithm in PDAC before CRT should be considered. According to the analysis of post-treatment quantitative PET parameters, decreases in SUV_max_ and SUV_mean_ might be associated with favorable local tumor control. Interestingly and contrary to preclinical data, radiotherapy did not seem to increase FAPI uptake as a correlate of radiation-induced fibrosis in our patient cohort. Despite the preliminary findings of the current study, the benefit of reducing the patient´s burden by sparing them from unnecessary therapy through advanced staging imaging is highlighted. Utilizing alternative diagnostic measures, tumor marker dynamics and symptom assessment can serve as a comprehensive approach to overcome the lack of histopathological confirmation for FAPI positive lesions not amenable for biopsy. Prospective longitudinal studies with larger patient cohorts are needed to validate the current explorative results and to verify the beneficial aspects of [^68^Ga]Ga-FAPI PET/CT and post-treatment PET metrics for improvement of long-term outcome after chemoradiation in patients with LAPAC and LRPAC.

## Supplementary Material

Supplementary tables.

## Ethical Approval Statement

This study was approved by the local institutional review board under the approval number EA1/241/22.

## Figures and Tables

**Table 1:** Patient characteristics. *LAPAC* locally advanced pancreatic adenocarcinoma, *LRPAC* locally recurrent pancreatic adenocarcinoma, *FOLFIRINOX* folinic acid, fluorouracil, irinotecan and oxaliplatin.

Patient demographics	
**Female, N (%)**	15 (55.6)
**Age, median (range)**	61 (42-89)
**Indication**	N (%)
LAPAC	12 (44.4)
LRPAC	15 (55.6)
**Tumor location**	N (%)
Head	21 (77.8)
Body	5 (18.5)
Tail	1 (3.7)
**Previous chemotherapy**	N (%)
FOLFIRINOX	15 (55.6)
Gemcitabine & nab-paclitaxel	3 (11.1)
FOLFIRINOX + Gem/nab-paclitaxel	8 (29.6)
Others	1 (3.7)
**Number of FAPI PET/CT follow-up scans**	N (%)
0	14 (51.9)
1	13 (48.1)
2	7 (25.9)
3	4 (14.8)

**Table 2:** Treatment modifications based on FAPI PET/CT findings. No radiotherapy due to “other reasons” include tumor infiltration of surrounding organs or no detection of measurable vital tumor tissue.

	Initial staging n = 27	First follow-up (3 months) n = 13	Second follow-up (6 months) n = 7	Third follow-up (9 months) n = 4
Additional diagnostic measures	1 (4%)	3 (23%)	2 (29%)	0
Altered radiotherapy volume due to cN1/2 to cN0 downstaging	8 (30%)	-	-	-
No radiotherapy due to cM1 upstaging	4 (15%)	-	-	-
No radiotherapy due to other reasons	2 (7%)	-	-	-
Follow-up: systemic chemotherapy or best supportive care due to cM1 upstaging	-	3 (23%)	2 (29%)	2 (50%)
Follow-up: ablative radiotherapy of cN1 and cM1 oligometastases	-	2 (15%)	1 (14%)	1 (25 %)
