# Supplementary material for: 68Ga-labeled fibroblast activation protein inhibitor (FAPI) PET/CT for locally advanced or recurrent pancreatic cancer staging and restaging after chemoradiotherapy
Source: Theranostics. 2024 Jul 8;14(11):4184–97. doi: 10.7150/thno.95329 (PMC11303068; doi:10.7150/thno.95329)
Supplement: Supplementary file 1 — Supplementary tables. [file thnov14p4184s1.pdf]

Patient	Age (y)	Sex	Tumor location	Previous surgery	Initial tumor classification	Grading	Previous chemotherapy	Clinical indication for RCTx
**1**	69	M	Head	Whipple	pT2 pN1 (2/12) cM0 L1 V0 Pn0 R0	G3	Gem+Cap	LRPAC
**2**	80	M	Head	Whipple	pT2 pN1 (1/25) L0 V0 Pn1 R1	G3	Gem	LRPAC
**3**	80	M	Head	No	cT3 cN0 cM0	G2	FOLFIRINOX<br>Gem+nab/Paclitaxel	LAPAC
**4**	60	W	Body	No	cT4 cN1 cM0	G2	FOLFIRINOX	LAPAC
**5**	42	M	Tail	Distal pancreatectomy	pT3 pN1(1/30) L1 V0 Pn1 R0	G3	FOLFIRINOX<br>Gem+nab/Paclitaxel	LRPAC
**6**	56	W	Head	pp-Whipple	pT3 pN0 (0/15) L0 V0 Pn1 R0	G3	FOLFIRINOX<br>Gem+nab/Paclitaxel	LRPAC
**7**	58	M	Head	Whipple	pT2 pN2 (13/30) L1 Pn1 R1	G3	FOLFIRINOX	LRPAC
**8**	61	M	Head	Whipple	pT2 pN0 (0/14) cM0 L0 V0 Pn1 R0	G3	FOLFIRINOX	LRPAC
**9**	58	W	Head	Whipple planned; abort of operation due to intraoperative detection of liver metastasis	cT4 cN0 cM0<br>(pM1 (hep) intraoperative)	G2	FOLFIRINOX<br>Gem+nab/Paclitaxel	LAPAC
**10**	74	W	Body	No	cT4 cN0 cM0	G3	FOLFIRINOX	LAPAC
**11**	64	M	Head	Abort of operation due vessel infiltration	cT2 cN0 cMx	G3	FOLFIRINOX	LAPAC
**12**	75	W	Body	Distal pancreatectomy	pT2 pN0 (0/4) L0 V0 Pn0 R0	G1	Gem+nab/Paclitaxel	LRPAC
**13**	57	M	Head	No	cT4 cN0 cM1 (hep)	high grade intraepithelial neoplasm	FOLFIRINOX	LAPAC

**14**	49	W	Head	Duodeno-pancreatectomy	pT3 pN1 (6/39) L0 V0 Pn0 R0	G2	Gem/Cis<br>Gem<br>FOLFIRINOX	LRPAC
**15**	62	M	Head	Whipple	pT2 pN1 (1/24) L0 V0 Pn1 R0	G2	FOLFIRINOX<br>Gem+nab/Paclitaxel	LRPAC
**16**	64	M	Head	Whipple	pT3 pN2 (5/20) L1 V0 Pn1 G3 R1	G3	mFOLFIRINOX	LRPAC
**17**	89	W	Head	No	cT4 cN0 cM0	G3	FOLFIRINOX	LAPAC
**18**	62	W	Head	Pancreatectomy	ypT1c ypN0(0/26) L0 V0 Pn1 R1 cM0	G2	Neoadjuvant<br>mFOLFIRINOX<br>Adjuvant<br>mFOLFIRINOX<br>Gem+nab/Paclitaxel	LRPAC
**19**	58	W	Body	Whipple	pT2 pN2(8/19) L1 V0 Pn1 R0 G2	G2	mFOLFIRINOX	LRPAC
**20**	82	M	Head	Whipple	pT3a pN1a (1/21) pL1 pV0 Pn0 R0	G2	Capecitabine	LRPAC
**21**	52	W	Head	No	cT4 cN0 cM0	G3	mFOLFIRINOX	LAPAC
**22**	64	W	Head	No	cT4 cN0 cM0	Borderline	FOLFIRINOX<br>Gem/Abraxane	LAPAC
**23**	54	W	Body	Whipple	pT1c pN1(1/19) L1 V0 Pn1 R0	G3	FOLFIRINOX	LRPAC
**24**	56	M	Head	Whipple	pT2 pN2(5/36) L1 V0 R0Pn1	G3	mFOLFIRINOX	LRPAC
**25**	61	W	Body	No	cT4 cN0 cM0	G3	FOLFIRINOX	LAPAC
**26**	57	M	Head	No	cT2 cN0 cM0	G2	mFOLFIRINOX	LAPAC
**27**	68	W	Head	No	cT4 cN2 cM0	G3	FOLFIRINOX	LAPAC

Supplementary Table S1. Detailed patient characteristics. *FOLFIRINOX* folinic acid, fluorouracil, irinotecan and oxaliplatin. *RTx* radiotherapy. *LAPAC* locally advanced pancreatic adenocarcinoma. *LRPAC* locally recurrent pancreatic adenocarcinoma.

Patient	Scan before radiotherapy/ follow-up	TNM (CT-based)	TNM (FAPI-PET/CT-based)	Additional findings in FAPI PET/CT	Staging change	Change of therapy after FAPI-PET/CT	Additional diagnostic measures
**1**	Before RTx	T4 N1 M0	T4 N1 M0	None	No	No	None
	1st follow-up	T0 N0 M0	T4 N0 M1 ((LYM)	Local, recurrent tumor tissue detectable, distant lymph node metastasis	Up	Radiation of distant lymph node metastasis	Biopsy of distant lymph node metastasis and confirmation PDAC
	2nd follow-up	T4 N0 M1(LYM)	T4 N1 M1(OTH,LYM)	Soft tissue metastasis	Up	Palliative intended radiation therapy of distant metastasis	None
	3rd Follow-up	T4 N0 M1(LYM,ADR,HEP)	T4 N0 M1(PER,HEP,BRA,ADR,OTH,LYM)	Diffuse metastases	Up	No (Best supportive care)	Cranial MRI
**2**	Before RTx	T1 N0 M0	T1 N0 M0	None	No	No	None
	1st follow-up	T0 N0 M0	T0 N0 M0	None	No	No	None
	2nd follow-up	T0 N0 M0	T0 N0 M0	None	No	No	None
	3rd follow-up	T0 N0 M0	T0 N0 M1(OSS)	Bone metastasis	Up	Radiation of Oligometastasis	None
**3**	Before RTx	T4 N1 M1(PER)	T4 N1 M1(HEP, PER)	None	No	No (No radiotherapy; systemic chemotherapy only)	None
**4**	Before RTx	T4 N1 M0	T4 N0 M0	Morphologically suspicious mesenterial lymph nodes without FAPI expression	Down	Change of target volume	None
	1st follow-up	T4 N1 M0	T4 N0 M1(PER)	Morphologically suspicious mesenterial lymph nodes without FAPI expression, peritoneal carcinomatosis	Up	No	Biopsy: no malignancy
	2nd follow-up	T4 N1 M0	T4 N0 M1 (PER,HEP)	Morphologically suspicious mesenterial lymph nodes without FAPI expression,	Up	Systemic chemotherapy	None

				peritoneal carcinomatosis, hepatic metastasis			
**5**	Before RTx	T4 N1 M0	T4 N0 M0	Morphologically suspicious mesenterial lymph nodes without FAPI expression	Down	Changed target volume	None
	1st follow-up	T4 N1 M1 (HEP)	T4 N0 M1 (PUL,HEP,PER)	Pulmonary metastases, peritoneal carcinomatosis	Up	No (Systemic molecular therapy)	None
**6**	Before RTx	T4 N2 M0	T4 N0 M0	Morphologically suspicious mesenterial lymph nodes without FAPI expression	Down	No (Abort of radiotherapy due to poor general condition)	None
**7**	Before RTx	T4 N0 M0	T1 N0 M0	None	Down	No	None
	1st follow-up	T4 N0 M0	T0 N0 M0	No vital tumor tissue	Down	No	None
	2nd follow-up	T4 N0 M0	T0 N0 M0	No vital tumor tissue	Down	No	None
	3rd follow-up	T4 N0 M0	T2 N0 M1(HEP)	Liver metastases	Up	Systemic chemotherapy	Abdominal MRI: confirmation of liver metastases
**8**	Before RTx	T0 N1 M1(HEP)	T2 N1 M1(HEP,OSS)	Bone metastasis	Up	No (No radiotherapy; systemic therapy only)	None
**9**	Before RTx	T4 N1 M1	T3 N0 M0	Morphologically suspicious mesenterial lymph nodes without FAPI expression	Down	No (Refusal of radiotherapy; continuation of CTx)	None
**10**	Before RTx	T4 N1 M0	T4 N0 M0	Morphologically suspicious mesenterial lymph nodes without FAPI expression	Down	Change of target volume	None
	1st follow-up	T4 N0 M0	T4 N1 M0	Lymph node metastasis	Up	Radiotherapy of singular lymph node metastasis	None
	2nd follow-up	T4 N1 M0	T4 N2 M0	Diffuse lymph node metastases	Up	Systemic chemotherapy	None
**11**	Before RTx	T0 N0 M0	T2 N0 M0	Vital tumor tissue	Up	Indication for local radiotherapy	None
	1st follow-up	T0 N0 M0	T2 N0 M0	Vital tumor tissue	Up	No	None
	2nd follow-up	T0 N1 M0	T3 N0 M0	Vital tumor tissue	Up	No	None

	3rd follow-up	T0 N1 M0	T3 N0 M0	Vital tumor tissue	Up	No	None
**12**	Before RTx	T4 N0 M0	T4 N0 M0	None	No	No	None
**12**	1st follow-up	T4 N0 M0	T4 N0 M1(per)	Peritoneal carcinomatosis	Up	Systemic chemotherapy	None
**13**	Before RTx	T4 N2 M0	T4 N0 M0	Morphologically suspicious mesenterial lymph nodes without FAPI expression	Up	Change of target volume	None
	1st follow-up	T4 N0 M0	T4 N0 M1(PER)	Peritoneal carcinomatosis	Up	Systemic chemotherapy	None
**14**	Before RTx	T4 N1 M1(ADR)	T4 N0 M0	No detection of distant metastases	Down	Indication for local radiotherapy	None
	1st follow-up	T4 N1 M1(ADR)	T4 N1 M1(HEP)	Liver metastases	No	Systemic chemotherapy	Abdominal MRI
**15**	Before RTx	T4 N1 M0	T4 N0 M0	Duodenal infiltration	Up	No radiotherapy due to local infiltration	Abdominal MRI
**16**	Before RTx	T4 N0 M0	T0 N0 M0	Suspicious locally recurrent tissue without FAPI expression	Down	No radiotherapy	None
**17**	Before RTx	T2 N1 M0	T3 N0 M0	Morphologically suspicious mesenterial lymph nodes without FAPI expression	Down	Change of target volume	None
	1st follow-up	T4 N1 M0	T3 N0 M0	Morphologically suspicious mesenterial lymph nodes without FAPI expression	Down	Change of target volume	None
	2nd follow-up	T4 N0 M1(PUL)	T3 N0 M1(PUL)	None	No	No (systemic chemotherapy)	None
**18**	Before RTx	T0 N0 M0	T1 N0 M0	Vital tumor tissue detectable	Up	Indication for radiotherapy	None
	1st follow-up	T4 N1 M0	T2 N0 M0	Morphologically suspicious mesenterial lymph nodes without FAP-expression	Down	No	None

**19**	Before RTx	T4 N2 M1(PUL)	T4 N1 M1(PER)	Peripancreatic lymph node metastasis, peritoneal carcinomatosis	No	No (no radiotherapy; systemic chemotherapy only)	None
**20**	Before RTx	T4 N1 M0	T4 N0 M0	Morphologically suspicious mesenterial lymph nodes without FAPI expression	Down	Change of target volume	None
	1st follow-up	T4 N1 M1(HEP)	T0 N0 M1(PER,HEP)	Peritoneal carcinomatosis, no local vital tumor tissue	Up	No (systemic chemotherapy only)	None
**21**	Before RTx	T4 N1 M0	T4 N0 M1(PER)	Duodenal infiltration, peritoneal carcinomatosis	Up	No radiotherapy; systemic chemotherapy only	None
**22**	Before RTx	T4 N0 M0	T1 N0 M0	None	Down	No	None
**23**	Before RTx	T4 N1 M0	T4 N0 M1(PER)	Peritoneal carcinomatosis	Up	No radiotherapy; systemic chemotherapy only	None
**24**	Before RTx	T4 N2 M0	T4 N0 M0	Morphologically suspicious mesenterial lymph nodes without FAPI expression	Down	Change of target volume	None
**25**	Before RTx	T4 N1 M0	T4 N0 M0	Morphologically suspicious mesenterial lymph nodes without FAPI expression	Down	Change of target volume	None
**26**	Before RTx	T4 N0 M0	T2 N0 M0	None	No	No	None
**27**	Before RTx	T4 N0 M0	T2 N0 M0	None	No	No	None

Supplementary Table S2. Detailed patient characteristics. Location of metastasis: *hep* hepatic, *per* peritoneal, *lym* lymphatic, *oss* bone, *adr* adrenal, *bra* brain. *RTx* radiotherapy
